# Differences in Self-Rated Memory by Race/Ethnicity

**DOI:** 10.1093/geroni/igab046.2106

**Published:** 2021-12-17

**Authors:** Yujin Franco, Joseph Saenz, Yuri Jang, Jessica Ho

**Affiliations:** 1 University of Southern California, University of Southern California, California, United States; 2 University of Southern California, Los Angeles, California, United States

## Abstract

Self-rated memory is an important dimension of well-being among older adults that has also been linked to cognitive impairment over the long term. However, few studies based on nationally-representative samples have examined differences in self-rated memory by race/ethnicity. This study explores differences in self-rated memory across non-Hispanic White, non-Hispanic Black, and Hispanic older adults in the United States. Data were drawn from the 2011 wave of the National Health and Aging Trends Study (NHATS). The sample consisted of older adults aged 65 and older (N=4,753 non-Hispanic Whites, N=1,442 non-Hispanic Blacks, and N=388 Hispanics). Logistic regression was used to examine the association between having poor/fair self-rated memory and race/ethnicity, controlling for socio-demographic characteristics (age, gender, education level, income, and marital status), chronic conditions (heart attack, hypertension, diabetes, stroke, and depressive symptoms), objective memory status, functional limitations (activities of daily living and instrumental activities of daily living), and other social and cultural factors (economic vulnerability, religious practice, and limited English proficiency). I find that non-Hispanic Blacks and Hispanics have significantly higher odds of reporting poor/fair self-rated memory than non-Hispanic Whites. Compared to non-Hispanic Whites, Blacks and Hispanics had 33% and 56% higher odds of reporting poor/fair self-rated memory, respectively, controlling for sociodemographic characteristics, chronic conditions, objective memory status, functional limitations, and social and cultural factors. These results provide evidence that understanding differences in self-rated memory across racial/ethnic groups may have important implications for health professionals, particularly in relation to conducting and interpreting cognitive screening assessments.

